# From financial scarcity to risk and time preferences: the role of executive functions

**DOI:** 10.3389/fpsyg.2026.1782444

**Published:** 2026-07-08

**Authors:** Jiaqi Huang, Haibo Zhu

**Affiliations:** Agricultural Information Institute, Chinese Academy of Agricultural Sciences, Beijing, China

**Keywords:** executive functions, financial scarcity, risk preference, rural China, time preference

## Abstract

**Introduction:**

Understanding how financial scarcity influences intertemporal decision-making is crucial for designing effective poverty-alleviation policies. However, the cognitive mechanisms underlying this relationship remain insufficiently explored. This study examines the correlational patterns through which financial scarcity relates to risk and time preferences, with a particular focus on the mediating role of executive functions, specifically planning and initiating, attention, and self-control and self-monitoring.

**Methods:**

Cross-sectional survey data were collected from 468 rural residents in two historically impoverished counties in Yunnan Province, China. Financial scarcity was assessed using the Psychological Inventory of Financial Scarcity (PIFS), while executive functions were measured with a culturally adapted form of the Amsterdam Executive Function Inventory. Risk and time preferences were assessed through a lottery choice experiment and a money-earlier-or-later allocation task, respectively. Structural equation modeling with bootstrapped mediation tests was employed for data analysis.

**Results:**

The results indicate that financial scarcity is significantly associated with lower scores across all three dimensions of executive functioning. Critically, planning and initiating ability emerged as a consistent indirect pathway, yet with a notable divergence across preference domains: higher financial scarcity was associated with lower planning capacity, which in turn was associated with lower risk aversion, whereas lower planning ability was associated with decreased future orientation.

**Discussion:**

These findings are consistent with a function-selective refinement of the bandwidth model of scarcity and reveal a counterintuitive pattern in risk-related decisions, suggesting that cognitive depletion may paradoxically reduce risk aversion rather than uniformly impairing prudent choice. The study highlights the importance of designing targeted cognitive-scaffolding interventions to support sound decision-making under conditions of financial constraint.

## 1 Introduction

Ending poverty is the first goal of the 2030 Agenda for Sustainable Development (SDGs). By the end of 2022, 8.4% of the world’s population still lived in extreme poverty (United Nations, 2024). Poverty, or more broadly, financial scarcity, greatly affects people’s lives and development. When financial resources are scarce, the impact extends beyond the realm of finances or material possessions, affecting individuals’ psychological well-being as well (Haushofer and Fehr, 2014). The psychological consequences, such as impeding executive functions and cognitive abilities (Dalton et al., 2020; Fehr et al., 2019; Mani et al., 2013), increasing depression and anxiety (Fitch et al., 2011; Sweet et al., 2013), are associated with a wide range of counterproductive economic behaviors, which can lead to poverty traps (Schilbach et al., 2016; Shah et al., 2012).

The scarcity theory, proposed by Mullainathan and Shafir (2013), provides a new frontier in understanding the causes of poverty traps: scarcity itself induces a scarcity mindset, subsequently compelling the poor to make suboptimal economic decisions. This theory posits three specific hypotheses: scarcity leads to attentional focus and neglect of other useful information (tunneling), causing overborrowing; scarcity induces trade-off thinking, resulting in more consistent consumption decisions; and scarcity reduces mental bandwidth (i.e., cognitive capacity and executive functions), subsequently increasing risk aversion and time discounting.

Recent comprehensive reviews have revealed inconsistencies in the empirical literature on scarcity and decision-making. In a systematic examination of scarcity research, De Bruijn and Antonides (2022) found that while financial scarcity consistently predicts increased time discounting, evidence regarding its relationship with risk aversion remains remarkably mixed: some studies report heightened risk aversion, others find no significant association, and still others observe increased risk-seeking under conditions of resource deprivation. Moreover, the proposed cognitive mechanisms underlying these effects—particularly the mental bandwidth hypothesis—have received only weak empirical support. These findings suggest that the theoretical predictions of scarcity theory may be more nuanced than originally proposed, and that contextual factors, measurement approaches, and population characteristics likely moderate the observed relationships.

A further limitation concerns the operationalization of mental bandwidth in existing research. Most studies have relied on measures of fluid intelligence or general cognitive ability (Dalton et al., 2020; Fehr et al., 2019; Mani et al., 2013), which may not capture the specific executive processes that scarcity theory implicates. Executive functions—the neurocognitive mechanisms through which individuals regulate thought, action, and emotion in service of goal-directed behavior (Diamond, 2013)—are theoretically distinct from general intelligence and may be particularly vulnerable to resource deprivation, as they rely heavily on prefrontal cortical function, which is sensitive to stress and physiological depletion.

The measurement of scarcity itself presents another important consideration. Much of the existing literature has employed experimental manipulations of temporary resource scarcity—for example, using “before-after-payday” designs (Carvalho et al., 2016; Lichand and Mani, 2020) or varying levels of monetary challenges (Mani et al., 2013) to create scarcity conditions. Such approaches may not adequately capture the chronic, persistent deprivation experienced by individuals in resource-constrained environments. Chronic scarcity, as opposed to acute experimental deprivation, may have qualitatively different effects on cognitive function due to the sustained physiological and psychological stress it engenders.

Motivated by these gaps and limitations, the present study examines the relationships among financial scarcity, executive functions, and risk and time preferences within a rural population in former nationally designated poverty counties in China. Rather than testing universal causal mechanisms, this study aims to document whether and how financial scarcity is associated with executive functions and economic preferences in this specific, understudied context. A growing body of research has investigated the cognitive and behavioral consequences of scarcity within Chinese populations. For instance, studies have examined the relationship between poverty and cognitive function among Chinese residents (Yu et al., 2021; Chen and Cao, 2020; Li et al., 2021), the impact of financial constraints on risk-taking among Chinese farmers (Liu et al., 2025), and the role of perceived scarcity on decision-making in urban Chinese samples (Zhu et al., 2025). What distinguishes our study from this existing work, beyond its theoretical aims, is its specific geographical and demographic focus. By concentrating on rural residents in former nationally designated poverty counties, individuals who are particularly likely to experience perceived scarcity, we provide novel evidence that complements and diversifies the current literature.

This study contributes to the literature in several ways. First, it examines the associations role of mental bandwidth in shapingwith risk and time preferences by distinguishing between cognitive capacity and executive functions. We emphasize executive functions because previous scarcity research has tended to focus on cognitive capacity—often operationalized as fluid intelligence—as a proxy for mental bandwidth, an approach not entirely consistent with the theoretical framework. Second, this study utilizes the Psychological Inventory of Financial Scarcity (PIFS), a scale measuring subjective perceptions of one’s financial situation and the affective and cognitive responses to these appraisals (van Dijk et al., 2022). Unlike studies that rely on short-term financial variations, this approach captures individuals’ experience of chronic scarcity over an extended period in real-life contexts. Third, by focusing on former nationally designated poverty counties in rural China, this study provides empirical evidence from an understudied population, thereby contributing geographical and demographic diversity to the growing body of literature on scarcity and decision-making.

The remainder of this paper is structured as follows: Section 2 reviews the relevant literature and develops the theoretical hypotheses. Section 3 describes the data sources, sample characteristics, and measurement of key variables. Section 4 presents the empirical results, including descriptive statistics and mediation analyses. Section 5 discusses the findings, outlines theoretical contributions and practical implications, acknowledges limitations, and suggests directions for future research.

## 2 Theoretical framework and hypotheses

### 2.1 Scarcity theory

This study adopts the psychological perspective offered by scarcity theory, as advanced by Mullainathan and Shafir (2013), who define the experience of scarcity as “having less than you feel you need” (p. 5). This definition underscores the subjective nature of scarcity, shifting focus from purely objective indicators such as income or social status toward an individual’s perceived shortfall. Such a subjective framing is critical for examining the psychological mechanisms through which scarcity operates.

According to Mullainathan and Shafir (2013), the experience of scarcity induces a “scarcity mindset,” which systematically influences cognition and behavior. Two central consequences are highlighted: tunneling, which refers to an intense attentional focus on the scarce resource to the neglect of other important considerations, and reduced mental bandwidth, reflecting a decline in overall cognitive capacity and executive functioning. Together, these cognitive effects are posited to lead to suboptimal decision-making—such as heightened impulsivity, increased temporal discounting, and a diminished ability to plan for future outcomes—even when such choices may contradict an individual’s long-term interests.

### 2.2 Financial scarcity and executive functions

According to scarcity theory, the experience of resource scarcity consumes finite cognitive resources, leading to a depletion of mental bandwidth (Mullainathan and Shafir, 2013). This cognitive tax is theorized to impair executive functions, a set of higher-order cognitive processes that include attentional control, planning and initiating, and self-control (Diamond, 2013). Empirical studies have generally supported this view. Research using both experimental and survey methods has demonstrated that financial scarcity is associated with deficits in attention (Zhang et al., 2023), and diminished self-control (Lichand and Mani, 2020; Yu et al., 2024). Using electroencephalography during a visual search task, Zhang et al. (2023) found that individuals primed with a scarcity mindset exhibited reduced selective attention. Lichand and Mani (2020) employed Stroop tests with Brazilian farmers and demonstrated that negative income shocks predicted lower attention and impulse control. More recently, Yu et al. (2024) conducted a cross-sectional online survey of college students and reported a negative correlation between perceived scarcity and self-control.

While attention and self-control have been more studied, empirical evidence on the effect of financial scarcity on planning and initiating remains scarce. This study extends this line of inquiry by examining these three core dimensions of executive functioning within a rural Chinese context.

### 2.3 Financial scarcity, executive functions, and risk preference

Empirical findings regarding the relationship between financial scarcity and risk preference remain inconclusive. While scarcity theory posits that cognitive load may heighten loss aversion and thus increase risk aversion (Schilbach et al., 2016), the evidence is mixed. Some studies report a positive link between scarcity and risk aversion (Ong et al., 2019). However, others find no significant association (Carvalho et al., 2016), and a third set of studies indicates that scarcity may even reduce risk aversion (Dalton et al., 2020). Critically, even when associations are found, cognitive load has seldom been established as a robust explanatory mechanism. In their comprehensive review, De Bruijn and Antonides (2022) conclude that evidence for cognitive capacity and executive functions as mediators is either absent or weak, with only limited support from studies like Ong et al. (2019) who reported a weak mediating effect of cognitive control. Their study measured cognitive control using a flanker task, a psychological test designed to evaluate attentional control by having participants concentrate on a central stimulus while ignoring surrounding distractors. Nevertheless, this metric addresses only attentional control, leaving other essential aspects of executive cognitive function unexamined.

The research gap is twofold: First, existing studies have typically specified mental bandwidth narrowly as fluid intelligence or cognitive capacity (Mani et al., 2013; Fehr et al., 2019), rather than examining the multi-dimensional construct of executive functions as originally theorized. Second, limited study to date has simultaneously tested all three core executive functions as parallel mediators, which is necessary to determine whether scarcity affects risk preference through a general bandwidth reduction or through function-specific pathways.

To reconcile these inconsistent results and address these gaps, the present study proposes that executive functions—conceptualized as three distinct yet interrelated dimensions (planning, attention, and self-control)—operate as mediating mechanisms. Specifically, financial scarcity may exert its influence on risk preference not through a direct pathway, but rather indirectly via its differential effects on planning and initiating, attention, and self-control, which represent the cognitive resources essential for deliberating uncertain future outcomes.

### 2.4 Financial scarcity, executive functions, and time preference

The evidence linking financial scarcity to time preference is more consistent, with studies demonstrating that scarcity increases temporal discounting, thereby strengthening preference for immediate rewards (Bartos et al., 2018; Carvalho et al., 2016; Cassidy, 2018; Ong et al., 2019). However, the theoretical explanation—that scarcity-induced bandwidth depletion impairs future-oriented thinking—has received limited empirical support as a mediating mechanism. While Ong et al. (2019) reported a weak mediating effect of cognitive control, who found that debt relief for low-income Singaporean households reduced present bias. Carvalho et al. (2016) and Cassidy (2018) found no significant mediation and proposed that the increased time discounting may cause by the poor’s adaption to changes in liquidity constrains.

Although the correlation between scarcity and present bias is empirically well established, the specific cognitive resources whose depletion drives this effect remain unidentified. Research in cognitive psychology has firmly established that executive functions, particularly planning and self-control, are critical for future-oriented thinking and delayed gratification (Basile and Toplak, 2015; Bayer and Osher, 2018). However, these insights have not been systematically integrated into scarcity research. The present study addresses this gap by examining whether financial scarcity is associated with specific executive resources—most notably planning capacity—and whether such resources are in turn associated with individuals’ capacity for future-oriented decision-making.

### 2.5 Research aims and hypotheses

**Figure 1:**
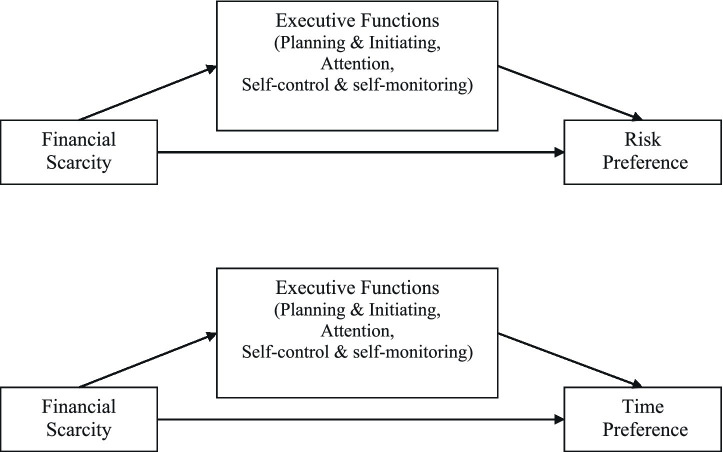
Hypotheses framework.

Integrating the theoretical and empirical perspectives reviewed above, this study aims to examine whether executive functions—conceptualized as planning and initiating, attention, and self-control and monitoring—are indirectly associated with the relationship between financial scarcity and two key domains of intertemporal preference: risk and time (see Figure 1). Specifically, the following hypotheses are proposed:

*Hypothesis 1 (H1)*: Executive functions are indirectly associated with the relationship between financial scarcity and risk preference. Higher levels of perceived financial scarcity are expected to be associated with lower executive function scores, which in turn are expected to be associated with higher risk aversion.

*Hypothesis 2 (H2)*: Executive functions are indirectly associated with the relationship between financial scarcity and time preference. Similarly, greater financial scarcity is predicted to be associated with lower executive function scores, which in turn are predicted to be associated with reduced future orientation (i.e., greater present bias).

By testing these hypotheses, the present research seeks to clarify the pathways through which subjective financial scarcity shapes economic decision-making, offering a more nuanced explanation for the inconsistent findings in the extant literature.

## 3 Method

### 3.1 Samples

This research draws on cross-sectional data collected through face-to-face surveys administered in March 2023 across 13 villages within two counties of Yunnan Province, China. These counties were originally classified as Nationally Defined Key Poverty Counties and were selected on the basis of their willingness to participate in the research. Households were randomly sampled from official local registration lists. Within each selected household, one individual was interviewed as the primary respondent, with priority given to the household head due to their central role in income generation and economic decision-making. When the household head was unavailable, another adult member of the household was interviewed instead. Consequently, the resulting sample comprised 468 rural residents (64.3% male), with household heads representing the majority of respondents (60.3%), followed by spouses (21.8%) and other household members (17.9%). The survey captured detailed information on perceived financial scarcity, executive functions, risk preference, time preference, income levels, and demographic characteristics.

### 3.2 Measures

#### 3.2.1 Financial scarcity

Financial scarcity was measured using the Psychological Inventory of Financial Scarcity (PIFS), a 12-item scale originally developed by van Dijk et al. (2022). The PIFS comprises four subdimensions, each assessed by three items: appraisal of insufficient financial resources, appraisal of low control over one’s financial situation, financial rumination and worry, and short-term focus. Example items are: “I often do not have enough money,” “I experience little control over my financial situation,” “I often worry about money,” and “I do not take future expenses into account.” (Full description for all 12 PIFS items are available in Supplementary Table S1). Respondents verbally rated their agreement on a 5-point Likert scale from 1 (totally disagree) to 5 (totally agree). An overall PIFS score was computed for each participant by averaging responses across all 12 items, with higher scores reflecting more pronounced perceived financial scarcity.

#### 3.2.2 Executive functions

Executive functions were measured using a shortened, culturally adapted version of the Amsterdam Executive Function Inventory (AEFI) (Van Der Elst et al., 2012). This scale originally covers three core domains—planning and initiating, attention, and self-control and self-monitoring—but to ensure comprehensibility among the surveyed rural residents, only five items were retained: two for planning and initiating (“I am well organized. For example, I am good at planning what I need to do during a day”; “It is easy for me to come up with a different solution if I get stuck when solving a problem”), one for attention (“I am easily distracted”), and two for self-control and self-monitoring (“I often react too fast after I’ve done or said something before it is my turn”; “I often forget what I have done yesterday”). Respondents indicated their agreement with each statement on a 3-point Likert scale ranging from 1 (not true) to 3 (completely true). After reverse-scoring relevant items, mean scores were computed separately for the two multi-item subscales (planning and initiating; self-control and self-monitoring). Higher scores reflect stronger executive functions in the corresponding domain.

We acknowledge that self-report measures of executive functions are less precise than objective behavioral tasks (e.g., Stroop task, Wisconsin Card Sorting Test, Trail Making Test). However, several practical considerations guided our choice. First, full behavioral assessments would have required 30–45 min per respondent, increasing fatigue and potentially reducing data quality. The abbreviated self-report format enabled efficient data collection without compromising participation rates. Second, given that our sample had limited formal education (*M* = 6.13 years), we were concerned that complex behavioral tasks might produce floor effects or confound performance with educational exposure rather than capturing genuine variation in executive functions. Self-report measures, while subject to introspective limitations, may be less vulnerable to such educational confounds in this population. Third, the AEFI has been validated in diverse international contexts, including populations with varying educational backgrounds (Van Der Elst et al., 2012; Escolano-Pérez et al., 2022). Nevertheless, we acknowledge the trade-offs inherent in this choice and address them in the limitations section.

#### 3.2.3 Translation and cultural adaptation procedure

The PIFS and AEFI were originally developed in English. To ensure cross-cultural validity and comprehensibility among rural Chinese respondents, we followed a rigorous translation-back translation procedure (Brislin, 1970). First, two independent bilingual researchers fluent in both English and Chinese translated the original instruments into Chinese. The two translations were compared, and any discrepancies were resolved through discussion to produce a consolidated Chinese version. Second, this consolidated version was back-translated into English by a third bilingual researcher who was blind to the original instruments. The back-translated versions were then compared with the originals to identify any semantic discrepancies or conceptual shifts. Third, the pre-final Chinese versions were pilot-tested with 30 rural residents from a village in Yunnan Province not included in the main study. During cognitive interviews following the pilot, respondents were asked to paraphrase each item in their own words to verify comprehension. Based on this feedback, minor wording adjustments were made to enhance clarity while preserving conceptual meaning. Finally, the finalized Chinese versions were reviewed by a panel of three experts in behavioral economics and rural development to confirm content validity.

#### 3.2.4 Risk preference

Risk preference is assessed through a lottery choice experiment, following the methodology of Barham et al. (2014), Ross et al. (2012), and Moore and Eckel (2003). Respondents are presented with a series of 11 choices between a certain payout and a gamble. The certain payoff is fixed at 10 yuan, while the outcome of the gamble depends on the color of a ball drawn from a bag with equal proportions of two colors (50/50). Each respondent is required to make all 11 decisions between a guaranteed payoff and an uncertain one (refer to the Supplementary Table S2). Upon completion of the experiment, respondents receive earnings based on one of their 11 decisions, randomly selected by choosing one card from 11 cards numbered correspondingly.

Respondents should switch at most once from the bet choice to the certain payout. They should also always choose the “Bet” option in the first row since in that row the certain payout is strictly dominated by the bet. Risk preference is quantified using the coefficient of relative risk aversion (CRRA) at the switching point of each respondent (Supplementary Table S2). For respondents who consistently choose the “Bet” option, a CRRA of −0.09 is assigned. A higher CRRA value indicates greater risk aversion.

#### 3.2.5 Time preference

Time preference was assessed using a money-earlier-or-later allocation task employing two incentivized multiple price lists, consistent with the approach of Angerer et al. (2015) and Fischer and Wollni (2018). Respondents were required to make trade-offs between receiving a smaller payoff earlier versus a larger payoff later. Each respondent was provided with 10 yuan and asked to allocate this amount between two dates – tomorrow and 2 weeks later. The money allocated to the later date was multiplied by a factor of 1.5. Consequently, the more money allocated to 2 weeks later, the greater the total amount received by the respondent (refer to the Supplementary Table S3). For instance, if a respondent chose to allocate 10 yuan to 2 weeks later and 0 yuan to tomorrow, they would receive 15 yuan in total 2 weeks later. Conversely, if they chose to allocate 0 yuan to 2 weeks later and 10 yuan to tomorrow, they would receive 10 yuan the next day. Respondents were informed that the payment they received from enumerators would match their allocation decision in terms of amount and date. Enumerators collected respondents’ contact information via WeChat, an instant communication mobile application widely used by 1.3 billion Chinese residents. Future payments were also distributed through WeChat.

The variable *t_i_* represents the amount of money allocated by a respondent to 2 weeks later, ranging from 0 to 10. This variable serves as a simple measure of the respondent’s future orientation and patience, with higher *t_i_* values indicating greater future orientation and patience.

#### 3.2.6 Control variables

A number of factors were controlled for in the analysis, including the respondent’s gender, years of schooling, age, the log of household per capita income, the area of land contracted by the household, and the county of residence. Gender was controlled for based on its established associations with the study’s core constructs. Previous research suggests women are more likely to experience financial distress than men (Zhou et al., 2024) and tend to be both more risk-averse and slightly less patient than men (Falk et al., 2018). Gender differences in executive functions, however, remain inconclusive (Grissom and Reyes, 2019). Years of schooling was included as educational attainment is closely tied to cognitive and decision-making abilities. Higher education is generally associated with stronger executive functions (Voos et al., 2011), lower risk aversion (Outreville, 2015), and greater future orientation (Redondo and Fierro, 2008). Age was controlled given that executive functions, risk preferences, and time preferences vary across the lifespan (Salthouse, 2019). While certain cognitive abilities may decline with age, older adults often exhibit better self-regulation and long-term planning, alongside significantly higher risk aversion compared to younger individuals (Falk et al., 2018). Per capita income was used to account for household economic status, as higher income generally reduces the likelihood of short-term financial behaviors driven by material constraints (Haushofer and Fehr, 2014). Including this variable helps isolate the psychological effect of scarcity from the influence of absolute financial resources. County of residence was controlled for to account for potential unobserved regional heterogeneity.

### 3.3 Data analysis

The hypotheses were tested using Structural Equation Modeling (SEM). Two parallel mediation models were specified to examine whether executive functions mediate the relationship between financial scarcity and each outcome variable. The first model tested the hypothesis that financial scarcity influences risk preference through its impact on executive functions (planning and initiating, attention, and self-control and self-monitoring). The second model tested the parallel hypothesis for time preference.

To ensure robust inference, all SEM analyses were estimated using maximum likelihood estimation with robust standard errors to account for potential heteroskedasticity. The significance of the proposed indirect effects was further assessed using non-parametric bootstrapping with 1,000 resamples. This method generates bias-corrected confidence intervals for the indirect pathways, providing a powerful test of mediation that does not rely on assumptions of normality. All analyses were conducted using Stata 15.1.

## 4 Results

### 4.1 Descriptive analysis

Table 1 presents the descriptive statistics for the primary study variables—including the Psychological Inventory of Financial Scarcity (PIFS), executive functions, risk and time preferences—as well as the control variables. The sample was predominantly male (64%), with an average age of 51.64 years (*SD* = 11.48) and a mean years of schooling of 6.13 years (SD = 3.30). The contracted land area per household averages 19.45 mu (approximately 1.30 hectares). The average per capita income in 2022 was 12,129.28 yuan (SD = 9,890.05), equivalent to approximately 1,803.32 US dollars, which corresponds to 60.2% of the national average for rural residents in China, reflecting the relative economic disadvantage of the sample.

**Table 1:** Descriptive statistics of the PIFS, execution functions, risk preference, time preference, and control variables (*n* = 468).

Variables	M	SD
PIFS (Cronbach’s alpha = 0.807; Raykov’s composite reliability coefficient = 0.803)	2.75	0.61
Planning and initiating	2.49	0.51
Attention	2.26	0.79
Self-control and self-monitoring	1.86	0.63
Risk preference	0.87	1.37
Time preference	5.75	4.83
Gender (1 = male)	0.64	0.48
Years of schooling	6.13	3.30
Age	51.64	11.48
Per capita income (yuan/year)	12129.28	9890.05
Land (mu, 1 mu = 1/15 hectares)	19.45	15.26
County (1 = County A)	60.68%	

The PIFS demonstrated good internal consistency, with a Cronbach’s alpha of 0.807 (Nunnally and Bernstein, 1994) and a Raykov’s composite reliability coefficient of 0.803 (Raykov, 1997). The mean PIFS score was 2.75 (SD = 0.61), exceeding values reported in studies of Dutch students (2.16) and entrepreneurs (1.96) (van Dijk et al., 2022), indicating a more pronounced experience of financial scarcity among the present sample. Items with the highest mean scores included “I often do not have enough money” (M = 3.48, SD = 1.06), “I am constantly wondering whether I have enough money” (M = 3.26, SD = 1.08), and “I often worry about money” (M = 3.14, SD = 1.11). Full descriptive statistics for all 12 PIFS items are available in Supplementary Table S1.

Regarding executive functions, self-control and self-monitoring scored lower on average (M = 1.86, SD = 0.63) compared to planning and initiating (M = 2.49, SD = 0.51) and attention (M = 2.26, SD = 0.79). This pattern aligns with findings from Spanish university students (Escolano-Pérez et al., 2022), but differs from studies of Dutch adolescents (Van Der Elst et al., 2012) and US/UK adults (van Dijk et al., 2022), where self-control and self-monitoring tended to be higher and attention lower.

The sample’s average CRRA coefficient for risk preference was 0.87 (SD = 1.37), indicating generally risk-averse tendencies. This result is consistent with estimates from experimental studies in India (Harrison et al., 2005), the US (Holt and Laury, 2002), and Zimbabwe (Barr, 2003). For time preference, the mean *t_i_* was 5.75 (SD = 4.83), which is moderately higher than values reported among farmers in Ghana (Fischer and Wollni, 2018), suggesting a relatively stronger future orientation and patience within the present sample.

The average risk preference CRRA in the sample is 0.87 (SD = 1.37), suggesting prevalent risk aversion. This magnitude is consistent with findings from experiments conducted in India (Harrison et al., 2005) and aligns with studies in the US (Holt and Laury, 2002) and Zimbabwe (Barr, 2003).

The average time preference *t_i_* in the sample is 5.75 (SD = 4.83), slightly higher than that reported in the study of farmers in Ghana (Fischer and Wollni, 2018), suggesting a higher level of future orientation and patience among respondents in this study.

### 4.2 Mediation analysis

To test the hypotheses that executive functions mediate the relationship between financial scarcity and intertemporal preferences (H1 for risk preference, H2 for time preference), a series of structural equation models with robust standard errors were estimated. Non-parametric bootstrap procedures with 1,000 replications were employed to derive robust confidence intervals for the indirect effects. Control variables included gender, education, age, log household income per capita, the area of land contracted by the household, and county. The estimation results for control variables from the mediation analysis are provided in Supplementary Tables S4, S5.

#### 4.2.1 Financial scarcity and executive functions

Consistent across all models, financial scarcity exhibited significant negative associations with all three executive functions. Specifically, higher financial scarcity was associated with diminished planning and initiating ability (*β* = −0.166, *p* = 0.003 for risk sample; *β* = −0.145, *p* = 0.001 for time sample), impaired attentional capacity (*β* = −0.276, *p* < 0.001; *β* = −0.244, *p* < 0.001), and reduced self-control and self-monitoring (*β* = −0.132, *p* = 0.032; *β* = −0.094, *p* = 0.060). These results confirm that financial scarcity imposes a substantial cognitive tax on core executive resources.

#### 4.2.2 Mediation for risk preference

As shown in Table 2, only planning and initiating ability demonstrated a consistent indirect association with risk aversion. Financial scarcity was associated with significantly lower planning and initiating ability (*β* = −0.166, *p* = 0.003). In contrast to H1, planning ability itself demonstrated a positive association with risk aversion (*β* = 0.368, *p* = 0.026), suggesting that individuals with stronger planning and initiating skills tend to be more risk-averse. The indirect effect was negative and marginally significant (*β* = −0.061, *p* = 0.069), which implies that scarcity diminishes risk aversion by impairing planning and initiating capacity. Bootstrap bias-corrected confidence intervals based on 1,000 resamples excluded zero (95% CI = [−0.144, −0.011]) and this finding was confirmed with 5,000 resamples (95% CI = [−0.147, −0.011]), indicating that the result is not an artifact of insufficient replication. As a supplementary test, a joint significance test (MacKinnon et al., 2002) was conducted: both the a-path (*p* = 0.003) and b-path (*p* = 0.026) were statistically significant at the 5% level, providing additional support for the presence of an indirect association. The total effect of financial scarcity on risk aversion was not statistically significant (*β* = −0.112, *p* = 0.430).

**Table 2:** Results of mediation analysis on risk preference.

Path	*β*	SE	*z*	*p*	95% CI
Planning & initiating					
Direct effect					
(a-path) financial scarcity → planning	−0.166	0.055	−3.02	0.003	[−0.274, −0.058]
(b-path) planning → risk aversion	0.368	0.165	2.23	0.026	[0.044, 0.693]
Financial scarcity → risk aversion	−0.051	0.145	−0.35	0.724	[−0.335, 0.233]
Indirect effect					
Financial scarcity → planning → risk aversion	−0.061	0.034	−1.82	0.069	[−0.127, 0.005]
Bootstrap percentile CI					[−0.135, −0.005]
Bootstrap bias-corrected CI					[−0.144, −0.011]
Total effect					
Financial scarcity → risk aversion	−0.112	0.142	−0.79	0.430	[−0.392, 0.167]
Attention					
Direct effect					
(a-path) financial scarcity → attention	−0.276	0.075	−3.69	0.000	[−0.423, −0.130]
(b-path) attention → risk aversion	0.092	0.103	0.89	0.372	[−0.110, 0.294]
Financial scarcity → risk aversion	−0.087	0.145	−0.60	0.549	[−0.371, 0.197]
Indirect effect					
Financial scarcity → attention → risk aversion	−0.025	0.029	−0.87	0.386	[−0.083, 0.032]
Bootstrap percentile CI					[−0.095, 0.036]
Bootstrap bias-corrected CI					[−0.105, 0.028]
Total effect					
Financial scarcity → risk aversion	−0.112	0.142	−0.79	0.430	[−0.392, 0.167]
Self−control					
Direct effect					
(a-path) financial scarcity → self-control	−0.132	0.061	−2.14	0.032	[−0.252, −0.011]
(b-path) self-control → risk aversion	−0.026	0.144	−0.18	0.856	[−0.309, 0.257]
Financial scarcity → risk aversion	−0.116	0.144	−0.81	0.420	[−0.398, 0.166]
Indirect effect					
Financial scarcity → self-control → risk aversion	0.003	0.022	0.16	0.875	[−0.039, 0.046]
Bootstrap percentile CI					[−0.046, 0.048]
Bootstrap bias-corrected CI					[−0.037, 0.053]
Total effect					
Financial scarcity → risk aversion	−0.112	0.142	−0.79	0.430	[−0.392, 0.167]

Attention and self-control did not function as significant mediators. Although financial scarcity was associated with significantly lower attentional capacity and self-control, neither attention (*β* = 0.092, *p* = 0.372) nor self-control (*β* = −0.026, *p* = 0.856) was significantly associated with risk aversion.

#### 4.2.3 Mediation for time preference

Parallel analyses for time preference yielded a similar pattern (Table 3). Again, only planning and initiating ability emerged as a consistent indirect pathway, partially supporting H2. Financial scarcity was associated with significantly lower planning and initiating ability, and lower planning and initiating ability was associated with less future orientation (i.e., greater present bias, *β* = 0.962, *p* = 0.041). The indirect effect was negative and marginally significant (*β* = −0.139, *p* = 0.087), with the bootstrap bias-corrected interval based on 1,000 resamples excluding zero (95% CI = [−0.355, −0.020]), and this finding was confirmed with 5,000 resamples (95% CI = [−0.353, −0.015]). The joint significance test also supported the indirect association (a-path: *p* = 0.001; b-path: *p* = 0.041). The total effect of financial scarcity on time preference was not statistically significant (*β* = 0.041, *p* = 0.916).

**Table 3:** Results of mediation analysis on time preference.

Path	*β*	*SE*	*z*	*p*	95% CI
Planning & initiating					
Direct effect					
(a-path) financial scarcity → planning	−0.145	0.044	−3.32	0.001	[−0.231, −0.059]
(b-path) planning → time preference	0.962	0.470	2.05	0.041	[0.041, 1.883]
Financial scarcity → time preference	0.180	0.394	0.46	0.647	[−0.591, 0.952]
Indirect effect					
Financial scarcity → planning → time preference	−0.139	0.082	−1.71	0.087	[−0.299, 0.020]
Bootstrap percentile CI					[−0.318, 0.005]
Bootstrap bias-corrected CI					[−0.355, −0.020]
Total effect					
Financial scarcity → time preference	0.041	0.390	0.11	0.916	[−0.724, 0.806]
Attention					
Direct effect					
(a-path) financial scarcity → attention	−0.244	0.058	−4.19	0.000	[−0.358, −0.130]
(b-path) attention → time preference	−0.140	0.304	−0.46	0.646	[−0.736, 0.457]
Financial scarcity → time preference	0.007	0.400	0.02	0.986	[−0.776, 0.790]
Indirect effect					
Financial scarcity → attention → time preference	0.034	0.079	0.43	0.667	[−0.121, 0.190]
Bootstrap percentile CI					[−0.113, 0.210]
Bootstrap bias-corrected CI					[−0.134, 0.182]
Total effect					
Financial scarcity → time preference	0.041	0.390	0.11	0.916	[−0.724, 0.806]
Self-control					
Direct effect					
(a-path) financial scarcity → self-control	−0.094	0.050	−1.88	0.060	[−0.191, 0.004]
(b-path) self-control → Time preference	−0.661	0.372	−1.78	0.076	[−1.391, 0.068]
Financial scarcity → time preference	−0.021	0.393	−0.05	0.958	[−0.790, 0.748]
Indirect effect					
Financial scarcity → self-control → time preference	0.062	0.049	1.27	0.204	[−0.034, 0.158]
Bootstrap percentile CI					[−0.019, 0.198]
Bootstrap bias-corrected CI					[−0.007, 0.216]
Total effect					
Financial scarcity → time preference	0.041	0.390	0.11	0.916	[−0.724, 0.806]

Attention and self-control did not function as significant mediators in this relationship. Although financial scarcity was associated with lower attention, attention itself was not significantly associated with time preference. Self-control and self-monitoring showed a marginally significant negative association with future orientation (*β* = −0.661, *p* = 0.076), indicating that individuals with greater self-control were less inclined toward future-oriented choices. Nevertheless, the indirect effect remained non-significant, as evidenced by bootstrap confidence intervals that included zero.

### 4.3 Summary

The mediation analyses reveal that financial scarcity is negatively associated with all three executive functions—planning and initiating, attention, and self-control and self-monitoring. However, only planning and initiating ability consistently shows an indirect association with intertemporal preferences, albeit with distinct patterns across domains. Financial scarcity is associated with lower planning and initiating capacity, which in turn is associated with lower risk aversion (contrary to H1) and is associated with lower future orientation (supporting H2). Attention and self-control, while associated with financial scarcity, do not show indirect associations with economic preferences in this sample.

## 5 Discussion

This study leverages field survey data from rural China to examine the relationships among financial scarcity, executive functions, and intertemporal preferences. It provides empirical evidence from a rural Chinese context, contributing to the geographically diverse body of literature on scarcity and decision-making.

### 5.1 The cognitive tax of scarcity: evidence and boundary conditions

Scarcity theory posits that scarcity captures mental bandwidth, thereby impairing cognitive capacity and executive function (Mullainathan and Shafir, 2013). The findings strongly support this proposition, demonstrating that financial scarcity negatively affects planning and initiating, attentional capacity, and self-control and self-monitoring.

This alignment with some studies (Mani et al., 2013; Ong et al., 2019; van Dijk et al., 2022) but contradiction with others (Fehr et al., 2022; Carvalho et al., 2016; Lichand and Mani, 2020) may stem from critical variations in the operationalization of scarcity. While some studies induce short-term variations (e.g., pre- vs. post-payday) (Mani et al., 2013), the present research employs self-reported measures capturing a chronic, subjective state of “having less than needed.” Short-term liquidity constraints may not equivalently diminish executive functions, as they may be offset by the certainty of imminent relief (Carvalho et al., 2016). Conversely, chronic scarcity or profound income uncertainty may be the primary drivers of the observed cognitive tax (Lichand and Mani, 2020). This study’s approach, similar to van Dijk et al. (2022), captures a persistent psychological state, offering insights arguably more aligned with real-world experiences of scarcity, despite the inherent limitations of cross-sectional data in establishing causality.

### 5.2 Mediating pathways to intertemporal preferences: a nuanced mechanism

Scarcity theory further suggests that financial scarcity increases risk aversion and time discounting via cognitive load (Schilbach et al., 2016). The current analysis, however, finds no significant direct associations between scarcity and either risk or time preferences. This absence of a direct effect is not uncommon; literature reviews note inconsistent evidence for scarcity increasing risk aversion, with almost no evidence for cognitive load as the underlying mechanism (De Bruijn and Antonides, 2022).

The pivotal contribution of this study lies in identifying a consistent indirect pathway via planning and initiating ability, although the statistical evidence is modest. The finding that better planning and initiating ability is associated with greater risk aversion represents a critical theoretical reversal. It aligns with emerging evidence that higher cognitive and executive functioning can correlate with more cautious choices (Andreoni et al., 2020; Trémeau et al., 2008; Olschewski et al., 2023). This challenges the simplistic equation of cognitive depletion with irrational risk-seeking. Instead, it supports a prudent evaluation mechanism: intact planning and initiating ability enables individuals to more thoroughly simulate and weigh potential future losses. When faced with risky choices, individuals with stronger planning capacity may engage in more elaborate mental simulation of adverse outcomes, leading to heightened loss aversion and consequently more risk-averse choices (Andreoni et al., 2020). From this perspective, risk aversion reflects not irrational fear but rather a sophisticated capacity to foresee and avoid potential losses. Scarcity, by eroding planning capacity, may lead to choices that appear less risk-averse due to a degraded ability to engage in such forward-looking, loss-averse deliberation.

While we have proposed a prudent evaluation mechanism to explain why reduced planning capacity is associated with lower risk aversion, several alternative explanations warrant careful consideration. First, the lottery choice task requires probabilistic reasoning and expected value calculations, and respondents must understand that the expected value of the gamble varies across the 11 choices and that switching from the gamble to the certain payoff at the appropriate point maximizes expected utility. If self-reported planning ability is conflated with numeracy or general cognitive ability, those with higher planning scores may simply understand the task better and make choices more consistent with expected value maximization—which, in this task, involves being relatively risk-averse. Conversely, those with lower planning scores may make more random choices due to task misunderstanding, which could manifest as lower risk aversion, suggesting that the observed relationship may reflect cognitive ability rather than planning-specific processes. Second, individuals experiencing poverty may have different reference points for what constitutes a “gain” or “loss” (Kahneman and Tversky, 1979). For those facing financial scarcity, the certain 10 yuan may be perceived as a substantial gain relative to their daily resources, rendering the risk-averse choice more attractive. For individuals not experiencing scarcity, the same 10 yuan may be less salient. If planning capacity correlates with resource levels—as our mediation model suggests—the observed differences in risk aversion could reflect differential valuation of the certain payoff rather than the direct effect of planning ability per se.

As for time preference, the finding aligns with the hypothesis and existing literature (Basile and Toplak, 2015; Bayer and Osher, 2018): the tendency to wait for a larger delayed reward on all of the time discounting measures was associated with higher executive functions.

It is also important to consider whether our measurement approach may have influenced the observed pattern of results. The finding that only planning and initiating (not attention or self-control) is associated with the scarcity-preference relationship could reflect differential validity of the self-report items. The planning items (“I am well organized,” “It is easy for me to come up with different solutions”) may tap more observable, consciously accessible behaviors, whereas attention and self-control items may require greater introspective accuracy or be more susceptible to social desirability bias. Alternatively, the null findings for attention and self-control could reflect true functional specificity, but we cannot rule out measurement-related explanations.

Although we found a marginal significant indirect pathway via planning and initiating ability, the total effects of financial scarcity on both risk and time preferences were not statistically significant. This pattern—significant indirect effects alongside non-significant total effects—can occur under several conditions. First, when effect sizes are modest, the total effect may not reach statistical significance due to limited power, while the more specific indirect pathway may still be detectable (Kenny and Judd, 2014). Second, in the time preference model, the direct and indirect effects had opposite signs (−0.139, +0.180), partially canceling each other and resulting in a near-zero total effect. These interpretations suggest that the absence of a significant total effect does not necessarily indicate the absence of a meaningful relationship, but rather points to the need for larger samples to estimate the total effect with greater precision.

### 5.3 Specificity of executive functions and clarifying mental bandwidth

The analysis reveals functional specificity among executive components. Although financial scarcity impaired all three measured functions, only planning and initiating consistently mediated its effects on economic preferences. Attention and self-control showed no significant mediating roles. This specificity underscores the need to move beyond the parsimonious but overly broad concept of “reduced mental bandwidth.” Competing effects may exist within this construct. For instance, while certain cognitive abilities (e.g., fluid intelligence) might negatively correlate with risk aversion (Andreoni et al., 2020), specific executive functions like planning and initiating may correlate positively. Future research may rigorously disentangle the definitions and differential impacts of various cognitive sub-components—such as cognitive capacity versus executive functions—on economic decision-making to refine scarcity theory.

### 5.4 Practical implications

The findings of this study inform targeted behavioral interventions. If financial scarcity primarily distorts intertemporal choices by eroding planning and initiating capacity, interventions should move beyond generic financial education or incentive-based approaches. Instead, they can adopt “cognitive scaffolding” strategies designed to compensate for impaired executive resources. In the domain of time preference, this might include automated commitment devices (e.g., default savings plans, round-up investment apps) that externalize the planning burden for long-term goals, structured decision aids that break down complex future trade-offs (e.g., retirement planning, educational investment) into simpler, sequential steps, and visualized future feedback (e.g., aging simulations, projected wealth curves) that make delayed consequences more salient and easier to process cognitively. Similarly, for risk preference, policies could simplify insurance or investment choices through intelligent defaults and reduced option complexity, provide planning prompts at key decision moments (e.g., before loan applications or large purchases) to encourage deliberate evaluation, and embed planning supports within financial products or advisory services. Such “scaffolded” choice architectures can help individuals with depleted bandwidth make decisions that better align with their long-term welfare.

### 5.5 Limitations and future directions

Several important limitations warrant acknowledgment. The cross-sectional nature of the data prevents firm conclusions regarding causality, and the measurement of executive functions relies on behavioral proxies rather than more direct cognitive or neuroscientific assessments. The self-report adaptation of the AEFI may introduce several potential biases. Self-reports of cognitive processes require introspection and accurate self-perception, which may be particularly challenging in populations with limited formal education. Social desirability bias may lead respondents to over report positive attributes like planning ability or underreport difficulties with self-control. Multi-method approaches combining self-reports, informant reports, and behavioral tasks would provide a more complete and robust assessment. Furthermore, the geographical specificity of the sample and the presence of attrition necessitate caution in generalizing the findings to broader populations.

The indirect effects reported in this study were only marginally significant at conventional levels, and the bootstrap bias-corrected confidence intervals’ upper bounds were close to zero. We have reported both 1,000 and 5,000 bootstrap resamples in the results, which yielded consistent confidence intervals, suggesting that the marginal significance reflects modest effect sizes rather than sampling instability. Consequently, these findings should be considered preliminary evidence, and replication in larger samples is necessary to confirm the robustness of the indirect pathways.

These limitations point to several productive avenues for future inquiry. Subsequent research could usefully explore the boundary conditions of the observed link between planning and initiating ability and risk aversion by examining how it varies across different risk domains and decision frames. Further investigation is also needed to assess whether attention and self-control might serve as significant mediators in other choice contexts not examined here. Finally, experimental or field-based studies that evaluate the efficacy of interventions designed to support or bolster planning capacity among populations experiencing financial scarcity would hold significant value for translating these theoretical insights into practical applications.

## 6 Conclusion

This study delineates correlational patterns consistent with a specific cognitive pathway linking financial scarcity and economic preferences, thereby offering nuance to our understanding of scarcity theory. The findings suggest that financial scarcity is associated with lower scores across all three executive functions, yet shows a selectively indirect association through planning and initiating ability. This indirect pathway is evident for both risk and time preferences, but in distinct ways. For risk preference, lower planning and initiating ability is associated with reduced risk aversion—a reversal of conventional expectations—which may reflect that scarcity is associated with reduced capacity for the deliberate, forward-looking evaluation that typically supports cautious choice. For time preference, diminished planning ability is associated with reduced future orientation, aligning with the hypothesized pathway and highlighting planning’s central role in intertemporal trade-offs. Recognizing this nuanced, function-specific pattern may inform the design of interventions that effectively address the cognitive roots of decision-making under financial constraint.

## Data Availability

The raw data supporting the conclusions of this article will be made available by the authors, without undue reservation.
