# Supplementary material for: From financial scarcity to risk and time preferences: the role of executive functions
Source: Front Psychol. 2026 Jul 8;17:1782444. doi: 10.3389/fpsyg.2026.1782444 (PMC13388038; doi:10.3389/fpsyg.2026.1782444)
Supplement: Supplementary file 1 [file Table_1.docx]

Supplementary Material

# Supplementary Tables

**Table S1. Descriptive statistics of the PIFS and its 12 items**

| Aspect | Item (1-12) | *M* | *SD* |
| --- | --- | --- | --- |
| An appraisal of insufficient financial resources | 1. I often don’t have enough money. | 3.48 | 1.06 |
|  | 2. I am often not able to pay my bills on time. | 2.46 | 1.07 |
|  | 3. I often don’t have money to pay for the things that I really need. | 2.98 | 1.07 |
| An appraisal of lack of control over one’s financial situation | 4. I experience little control over my financial situation. | 2.43 | 1.02 |
|  | 5. I think I am not able to manage my finances properly. | 2.38 | 1.01 |
|  | 6. When I think about my financial situation, I feel powerless. | 2.69 | 1.06 |
| Financial rumination and worry | 7. I am constantly wondering whether I have enough money. | 3.26 | 1.08 |
|  | 8. I have a hard time thinking about things other than my financial situation. | 2.59 | 1.03 |
|  | 9. I often worry about money. | 3.14 | 1.11 |
| A short-term focus | 10. I am only focusing on what I have to pay at this moment rather than my future expenses. | 2.53 | 1.04 |
|  | 11. I don’t take future expenses into account. | 2.48 | 1.10 |
|  | 12. Because of my financial situation, I live from day to day. | 2.52 | 1.19 |
|  | PIFS | 2.75 | 0.61 |

Note: PIFS is developed by van Dijk et al. (2022).

**Table S2. Experiment for measuring risk preference**

|  | Certain payout  (Yuan) | Bet | | Decision | CRRA |
| --- | --- | --- | --- | --- | --- |
|  |  | White Ball (50%)  (Yuan) | Yellow Ball (50%)  (Yuan) | 1= Bet  2= Certain payout |  |
| 1 | 10 | 20 | 10 | _____ | ∞ |
| 2 | 10 | 20 | 8 | _____ | 3.76 |
| 3 | 10 | 20 | 6.5 | _____ | 1.86 |
| 4 | 10 | 20 | 5 | _____ | 1.00 |
| 5 | 10 | 20 | 4 | _____ | 0.65 |
| 6 | 10 | 20 | 3.5 | _____ | 0.52 |
| 7 | 10 | 20 | 3 | _____ | 0.40 |
| 8 | 10 | 20 | 2.5 | _____ | 0.31 |
| 9 | 10 | 20 | 2 | _____ | 0.22 |
| 10 | 10 | 20 | 1 | _____ | 0.09 |
| 11 | 10 | 20 | 0 | _____ | 0.00 |

**Table S3. Experiment for measuring time preference**

| **Money Allocation choice:** | | | |
| --- | --- | --- | --- |
| Tomorrow: _____Yuan Two weeks later: _____Yuan | | | |
| **Allocation choices** | | **Money Received** | |
| Tomorrow | Two weeks later | Tomorrow | Two weeks later |
| 0 | 10 | 0 | 15 |
| 1 | 9 | 1 | 13.5 |
| 2 | 8 | 2 | 12 |
| 3 | 7 | 3 | 10.5 |
| 4 | 6 | 4 | 9 |
| 5 | 5 | 5 | 7.5 |
| 6 | 4 | 6 | 6 |
| 7 | 3 | 7 | 4.5 |
| 8 | 2 | 8 | 3 |
| 9 | 1 | 9 | 1.5 |
| 10 | 0 | 10 | 0 |

**Table S4. Control variable estimates for risk preference mediation models**

| **Variable** | **Planning and Initiating** | **Attention** | **Self-control and**  **self-monitoring** |
| --- | --- | --- | --- |
| Gender (1=Male) | 0.087 (0.066) | 0.079 (0.107) | 0.154 (0.079)† |
| Years of schooling | −0.094 (0.010) | −0.100 (0.017) | 0.010 (0.014) |
| Age | −0.030 (0.003) | −0.064 (0.005) | −0.128 (0.003)** |
| Log of per capita income | 0.111 (0.041) | −0.021 (0.064) | −0.031 (0.047) |
| Land | 0.086 (0.002) | 0.049 (0.004) | 0.126 (0.003)** |
| County (1=County A) | 0.139 (0.063)* | 0.097 (0.104) | −0.024 (0.079) |
| **Dependent Variable: Risk preference** | |  |  |
| **Variable** | **Planning and Initiating Model** | **Attention Model** | **Self-control and**  **self-monitoring Model** |
| Gender (1=Male) | −0.002 (0.180) | 0.005 (0.180) | 0.011 (0.181) |
| Years of schooling | 0.059 (0.029) | 0.052 (0.029) | 0.047 (0.029) |
| Age | 0.120 (0.008)† | 0.120 (0.008)† | 0.117 (0.008)† |
| Log of per capita income | −0.062 (0.114) | −0.046 (0.112) | −0.049 (0.111) |
| Land | −0.104 (0.005)* | −0.094 (0.005)† | −0.091 (0.005)† |
| County (1=County A) | 0.019 (0.179) | 0.032 (0.179) | 0.038 (0.177) |

Note: Cells show standardized coefficients with robust standard errors in parentheses. † *p* < .10, * *p* < .05, ** *p* < .01, *** *p* < .001.

**Table S5. Control variable estimates for time preference mediation models**

| **Variable** | **Planning and Initiating** | **Attention** | **Self-control and self-monitoring** |
| --- | --- | --- | --- |
| Gender (1=Male) | 0.034 (0.053) | 0.062 (0.079) | 0.185 (0.063)*** |
| Years of schooling | −0.047 (0.007) | −0.083 (0.013) | 0.031 (0.011) |
| Age | −0.054 (0.002) | −0.083 (0.004) | −0.116 (0.003)* |
| Log of per capita income | 0.021 (0.030) | 0.035 (0.047) | −0.017 (0.036) |
| Land | 0.061 (0.002) | 0.012 (0.003) | 0.065 (0.002) |
| County (1=County A) | 0.058 (0.052) | 0.145 (0.081)** | −0.012 (0.064) |
| **Dependent Variable: Time preference** | |  |  |
| **Variable** | **Planning and Initiating Model** | **Attention Model** | **Self-control and self-monitoring Model** |
| Gender (1=Male) | 0.025 (0.481) | 0.024 (0.486) | 0.039 (0.492) |
| Years of schooling | −0.009 (0.078) | 0.001 (0.078) | 0.006 (0.077) |
| Age | 0.108 (0.022)* | −0.115 (0.022)* | −0.123 (0.022)* |
| Log of per capita income | 0.048 (0.287) | −0.047 (0.291) | −0.050 (0.289) |
| Land | −0.011 (0.018) | 0.017 (0.018) | 0.022 (0.018) |
| County (1=County A) | 0.150 (0.480)** | −0.141 (0.492)** | −0.145 (0.485)** |

Note: Cells show standardized coefficients with robust standard errors in parentheses. † *p* < .10, * *p* < .05, ** *p* < .01, *** *p* < .001.
